# Association of Self-reported Presenting Symptoms With Timeliness of Help-Seeking Among Adolescents and Young Adults With Cancer in the BRIGHTLIGHT Study

**DOI:** 10.1001/jamanetworkopen.2020.15437

**Published:** 2020-09-03

**Authors:** Minjoung M. Koo, Georgios Lyratzopoulos, Annie Herbert, Gary A. Abel, Rachel M. Taylor, Julie A. Barber, Faith Gibson, Jeremy Whelan, Lorna A. Fern

**Affiliations:** 1 Epidemiology of Cancer and Healthcare Outcomes Research Group, Department of Behavioural Sciences and Health, University College London, London, United Kingdom; 2 Medical Research Council Integrative Epidemiology Unit, Bristol Medical School, University of Bristol, Bristol, United Kingdom; 3 Population Health Sciences, Bristol Medical School, University of Bristol, Bristol, United Kingdom; 4 University of Exeter Medical School, St Luke’s Campus, Exeter, United Kingdom; 5 Centre for Nurse, Midwife, and Allied Health Professional-led Research, University College London Hospitals NHS Foundation Trust, London, United Kingdom; 6 Department of Statistical Science, University College London, London, United Kingdom; 7 Centre for Outcomes and Experience Research in Children’s Health, Illness and Disability, Great Ormond Street Hospital for Children NHS Foundation Trust, London, United Kingdom; 8 School of Health Sciences, University of Surrey, Guildford, United Kingdom; 9 Cancer Division, University College London Hospitals NHS Foundation Trust, London, United Kingdom

## Abstract

**Importance:**

Evidence regarding the presenting symptoms of cancer in adolescents and young adults can support the development of early diagnosis interventions.

**Objective:**

To examine common presenting symptoms in adolescents and young adults aged 12 to 24 years who subsequently received a diagnosis of cancer and potential variation in time to help-seeking by presenting symptom.

**Design, Setting, and Participants:**

This multicenter study is a cross-sectional analysis of the BRIGHTLIGHT cohort study, which was conducted across hospitals in England. Participants included adolescents and young adults aged 12 to 24 years with cancer. Information on 17 prespecified presenting symptoms and the interval between symptom onset and help-seeking (the patient interval) was collected through structured face-to-face interviews and was linked to national cancer registry data. Data analysis was performed from January 2018 to August 2019.

**Exposures:**

Self-reported presenting symptoms.

**Main Outcomes and Measures:**

The main outcomes were frequencies of presenting symptoms and associated symptom signatures by cancer group and the proportion of patients with each presenting symptom whose patient interval was longer than 1 month.

**Results:**

The study population consisted of 803 adolescents and young adults with valid symptom information (443 male [55%]; 509 [63%] aged 19-24 years; 705 [88%] White). The number of symptoms varied by cancer group: for example, 88 patients with leukemia (86%) presented with 2 or more symptoms, whereas only 9 patients with melanoma (31%) presented with multiple symptoms. In total, 352 unique symptom combinations were reported, with the 10 most frequent combinations accounting for 304 patients (38%). Lump or swelling was reported by more than one-half the patients (419 patients [52%; 95% CI, 49%-56%]). Other common presenting symptoms across all cancers were extreme tiredness (308 patients [38%; 95% CI, 35%-42%]), unexplained pain (281 patients [35%; 95% CI, 32%-38%]), night sweats (192 patients [24%; 95% CI, 21%-27%]), lymphadenopathy (191 patients [24%; 95% CI, 21%-27%]), and weight loss (190 patients [24%; 95% CI, 21%-27%]). The relative frequencies of presenting symptoms also varied by cancer group; some symptoms (such as lump or swelling) were highly prevalent across several cancer groups (seen in >50% of patients with lymphomas, germ cell cancers, carcinomas, bone tumors, and soft-tissue sarcomas). More than 1 in 4 patients (27%) reported a patient interval longer than 1 month; this varied from 6% (1 patient) for fits and seizures to 43% (18 patients) for recurrent infections.

**Conclusions and Relevance:**

Adolescents and young adults with cancer present with a broad spectrum of symptoms, some of which are shared across cancer types. These findings point to discordant presenting symptom prevalence estimates when information is obtained from patient report vs health records and indicate the need for further symptom epidemiology research in this population.

## Key Points

**Question:**

What are the common presenting symptoms of cancer in adolescents and young adults aged 12 to 24 years and how are they associated with help-seeking?

**Findings:**

This cross-sectional analysis of 803 adolescents and young adult patients found a range of presenting symptoms in diverse combinations, which varied by cancer group; 27% of patients waited more than 1 month to seek help for their symptoms.

**Meaning:**

These findings suggest that adolescents and young adults with cancer present with a broad spectrum of symptoms, often at substantially higher proportions than previously reported; thus, re-examination of symptom prevalence in this patient group is warranted.

## Teaser

This cross-sectional study examines the common presenting symptoms of cancer in adolescents and young adults aged 12 to 24 years and the potential variation in time to help-seeking by presenting symptom.

## Introduction

Cancer among adolescents and young adults is rare but remains the most common cause of nonaccidental death in high-income countries.^1^ There are no effective interventions to support asymptomatic detection through population screening for most of this group, but expediting the diagnosis of individuals with symptoms may help improve clinical and patient-reported outcomes.^2,3,4,5,6,7,8^

Adolescents and young adults often experience prolonged intervals between onset of symptoms and diagnosis, more so than older adults with cancer.^9,10,11,12^ A critical component of overall diagnostic timeliness is the time between symptom onset and presentation, also known as the patient interval.^13^ Public health education campaigns are increasingly used as part of early diagnosis strategies to shorten this interval by promoting timely help-seeking.^14,15,16,17^ Indeed, improving symptom awareness has been identified as a research priority to improve early diagnosis for adolescents and young adults with cancer.^18^

Studying the presenting symptoms of adolescents and young adult cancers can help us understand time to help-seeking and associated variation among adolescents and young adults with symptoms who subsequently receive a diagnosis of cancer. However, there is limited evidence regarding the presenting symptoms of adolescents and young adults with cancer. Prior research^17,19,20^ is mostly based on clinician-recorded information contained in electronic health records (EHRs). Patient-reported data could amplify our understanding of presenting symptoms as approaches based on the examination of patient EHRs rely on complete and accurate elicitation and recording of symptom history during clinical encounters.^21^ Therefore, we aimed to examine the nature and frequency of presenting symptoms and to describe their associated time to help-seeking in adolescents and young adults with a subsequent diagnosis of cancer, using novel self-reported data from a large patient cohort in England.

## Methods

### Data and Study Population

The BRIGHTLIGHT study was approved by the London-Bloomsbury Research Ethics Committee and the National Health Service Health Research Authority Confidentiality Advisory Group. Participants gave written informed consent to take part in a face-to-face survey and for clinical information to be extracted from their EHRs; dates of diagnosis were obtained from national cancer registration (curated by Public Health England National Cancer Registration and Analysis Service). Participants younger than 16 years gave their assent in order to participate after consent was obtained from their parents or guardians. Those unable to complete the survey, unable to give consent, in custodial care, or facing imminent death were not eligible to take part in the study.^22^ This study follows the Strengthening the Reporting of Observational Studies in Epidemiology (STROBE) reporting guideline.

We analyzed cross-sectional patient-level data from the BRIGHTLIGHT cohort.^22^ A total of 1114 young people aged 12 to 24 years at diagnosis with any cancer were recruited between July 1, 2012, and April 30, 2015, from 96 nationwide English National Health Service Trust hospitals; 830 participants completed the baseline survey.

Information on events and intervals between symptom onset and cancer diagnosis was collected through a structured face-to-face interview conducted with an independent survey provider (Ipsos MORI) using a device with computer-assisted personal interviewing software, which allows the interviewer to input data into a tablet. This has the benefit of having built-in methods for checking errors and allows routing of questions, automatically eliminating irrelevant questions on the basis of previous answers. The survey instrument development has been described previously^23^; the survey consists of 15 domains identified by young people as important in their cancer experience, including their experience before diagnosis. The survey questions were mainly read out by the interviewer and answered from a prespecified list, with options for including free text and dates where applicable.

### Variables of Interest

Information on symptoms experienced before the diagnosis of cancer was ascertained through yes-or-no responses to 16 specified symptoms (patients could respond yes to >1 symptoms), with any other symptoms recorded as free-text responses.^23^ Symptoms derived from the free-text information were either recoded as 1 of the 16 specified symptoms, or otherwise collated into a 17th symptom category labeled as *other symptoms* (eTable 1 in the Supplement). Patients for whom symptom information was missing (10 patients), not specified (14 patients), or invalid (3 patients) were excluded from analyses (eFigure in the Supplement).

The patient interval was defined as the time from symptom onset to first presentation to the general practitioner or accident and emergency based on responses to the structured questionnaire.^12^ Participants were asked, “From the time when you first noticed a symptom of cancer how long was it before you saw the [general practitioner or accident and emergency]?” and they were provided with 1 of the following 6 response options: less than 1 week; 1 week up to 2 weeks; more than 2 weeks up to 4 weeks; more than 1 month up to 3 months; more than 3 months up to 6 months; or more than 6 months up to 12 months. We aggregated response categories as more than 2 weeks, more than 1 month, or more than 3 months.^12^

Gender, age at diagnosis, and residential postal code (matched to Local Super Output Area and used to derive Index of Multiple Deprivation 2015 scores), were extracted from case report forms, whereas information on ethnicity, relationship status, and employment status was elicited through the survey via self-report.^23^ Information on cancer type was extracted from cancer registry data. There were 70 patients (8%) for whom information could not be obtained from the cancer registry; for these patients, cancer site was taken from the self-completed survey response and cross-validated against the extracted clinical information from case report forms.

Cancers were grouped into 9 categories using the morphology-based classification of Birch et al^24^ for cancers common among the adolescent and young adult population according to their diagnoses as coded by the *International Statistical Classification of Diseases and Related Health Problems, Tenth Revision*. The 9 categories were as follows: leukemia; lymphoma; central nervous system and other intracranial and intraspinal neoplasms (central nervous system tumors); osseous and chondromatous neoplasms, Ewing tumor, and other neoplasms of bone (bone tumors); soft tissue sarcomas; germ cell and trophoblastic neoplasms (germ cell cancers); melanoma and skin carcinoma; carcinomas (except of the skin); and miscellaneous specified neoplasms not elsewhere classified and unspecified malignant neoplasms not elsewhere classified (grouped as unclassified or unspecified).

### Supplementary Analyses

We described the proportion of patients with a patient interval of more than 1 month by presenting symptom, stratified by cancer site (eTable 3 in the Supplement). For this analysis, we used the binary category of 1 month or less vs more than 1 month as examined previously.^12^

### Statistical Analysis

We calculated the frequency of presenting symptoms among the BRIGHTLIGHT cohort and described the resulting number of symptoms and symptom signature (ie, the nature and frequency of different symptoms) associated with each cancer group. Subsequently, we described proportions of patients with longer patient intervals by presenting symptom using the aforementioned 3 aggregated categories: more than 2 weeks, more than 1 month, and more than 3 months. All analyses were conducted using STATA SE statistical software version 15.1 (StataCorp). Data analysis was performed from January 2018 to August 2019.

## Results

### Frequency of Presenting Symptoms

Among 803 adolescents and young adults with valid information on symptoms, 443 (55%) were male, 509 (63%) were aged 19 to 24 years, and 705 (88%) described their ethnicity as White, as detailed previously.^12^ Lump or swelling was the most common symptom (reported by 419 patients [52%; 95% CI, 49%-56%]), followed by extreme tiredness (308 patients [38%; 95% CI, 35%-42%]) and unexplained pain (281 patients [35%; 95% CI, 32%-38%]). Night sweats (192 patients), lymphadenopathy (191 patients), and weight loss (190 patients) each were reported by almost one-quarter of patients before diagnosis (24%; 95% CI, 21%-27% for all). Seven of the 17 symptoms were reported by less than 10% of the study population (Table 1).

**Table 1.** Frequency of Presenting Symptoms Among the BRIGHTLIGHT Cohort

Symptom	Patients, No. (%) [95% CI] (N = 803)^a^
Lump or swelling	419 (52) [49-56]
Extreme tiredness	308 (38) [35-42]
Unexplained pain	281 (34) [32-38]
Night sweats	192 (24) [21-27]
Lymphadenopathy	191 (24) [21-27]
Weight loss	190 (24) [21-27]
Headaches	127 (16) [13-19]
Dizziness	126 (16) [13-18]
Rash or itching	94 (12) [10-14]
Limping or mobility problems	77 (10) [8-12]
Bruising or bleeding	73 (9) [7-11]
Other symptoms^b^	69 (9) [7-11]
Menstrual changes	56 (7) [5-9]
Recurrent infections	47 (6) [4-8]
Mole changes	40 (5) [4-7]
Loss of vision	27 (3) [2-5]
Fits or seizures	17 (2) [1-3]

^a^ Total sum exceeds 803 (100%) because patients could report multiple symptoms.

^b^ Other symptoms include symptoms with less than 2% frequency (see eTable 1 in the Supplement).

### Number of Presenting Symptoms

**Figure 1.**
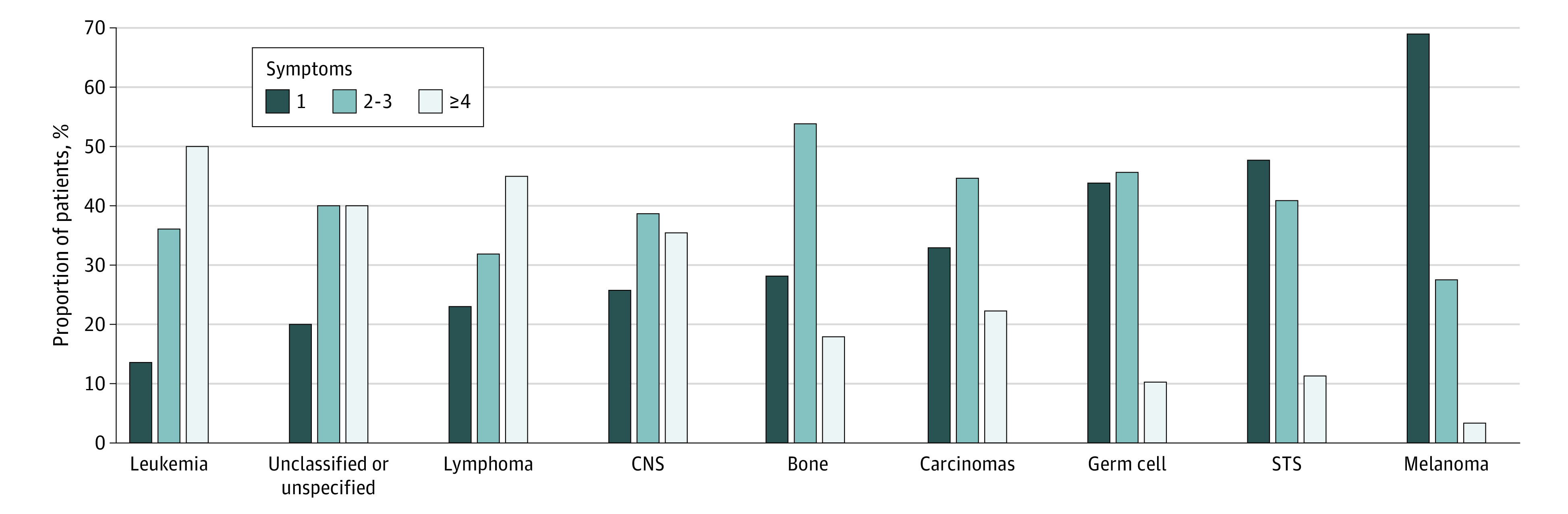
Number of Symptoms Reported by Adolescent and Young Adult Cancer Patients, by Cancer Type Symptoms are ordered by proportion of patients reporting a single symptom. CNS indicates central nervous system and other intracranial and intraspinal neoplasms; STS, soft-tissue sarcomas.

Overall, there was substantial patient-level variation in symptoms experienced before cancer diagnosis, with 557 patients (69%) reporting multiple symptoms. Patients with leukemia (88 patients [86%]) and lymphoma (200 patients [77%]) were most likely to report multiple symptoms, whereas those with melanoma were least likely to report multiple symptoms (9 patients [31%]) (Figure 1 and Table 2). In total, 352 unique symptom combinations were reported, with the 10 most frequent combinations accounting for 304 patients (38%).

**Table 2.** Proportion of Adolescent and Young Adult Cancer Patients With Different Patient Interval Lengths, by Symptom^a^

Symptom	Patients, total No. (N = 745)	Patients, No. (%), by interval		
		>2 wk	>1 mo	>3 mo
Recurrent infections	42	25 (60)	18 (43)	9 (21)
Loss of vision	21	15 (71)	8 (38)	3 (14)
Mole changes	40	25 (63)	15 (38)	10 (25)
Night sweats	179	103 (58)	64 (36)	28 (16)
Weight loss	177	94 (53)	57 (32)	28 (16)
Headaches	114	68 (60)	36 (32)	14 (12)
Menstrual changes	47	30 (64)	14 (30)	4 (9)
Unexplained pain	260	124 (48)	77 (30)	33 (13)
Extreme tiredness	282	150 (53)	83 (29)	39 (14)
Lymphadenopathy	181	89 (49)	50 (28)	22 (12)
Rash or itching	87	48 (55)	24 (28)	12 (14)
Bruising or bleeding	62	31 (50)	17 (27)	3 (5)
Dizziness	117	63 (54)	32 (27)	13 (11)
Lump or swelling	391	189 (48)	103 (26)	44 (11)
Limping or mobility problems	69	34 (49)	18 (26)	7 (10)
Other symptoms	66	29 (44)	17 (26)	6 (9)
Fits or seizures	17	6 (35)	1 (6)	1 (6)
All patients	745	358 (48)	204 (27)	91 (12)

^a^ Symptoms are ordered by proportion of patients with an interval between symptom onset and diagnosis (ie, patient interval) of longer than 1 month; columns do not sum to 745 because most patients had multiple symptoms.

### Cancer Site–Specific Symptom Signatures

**Figure 2.**
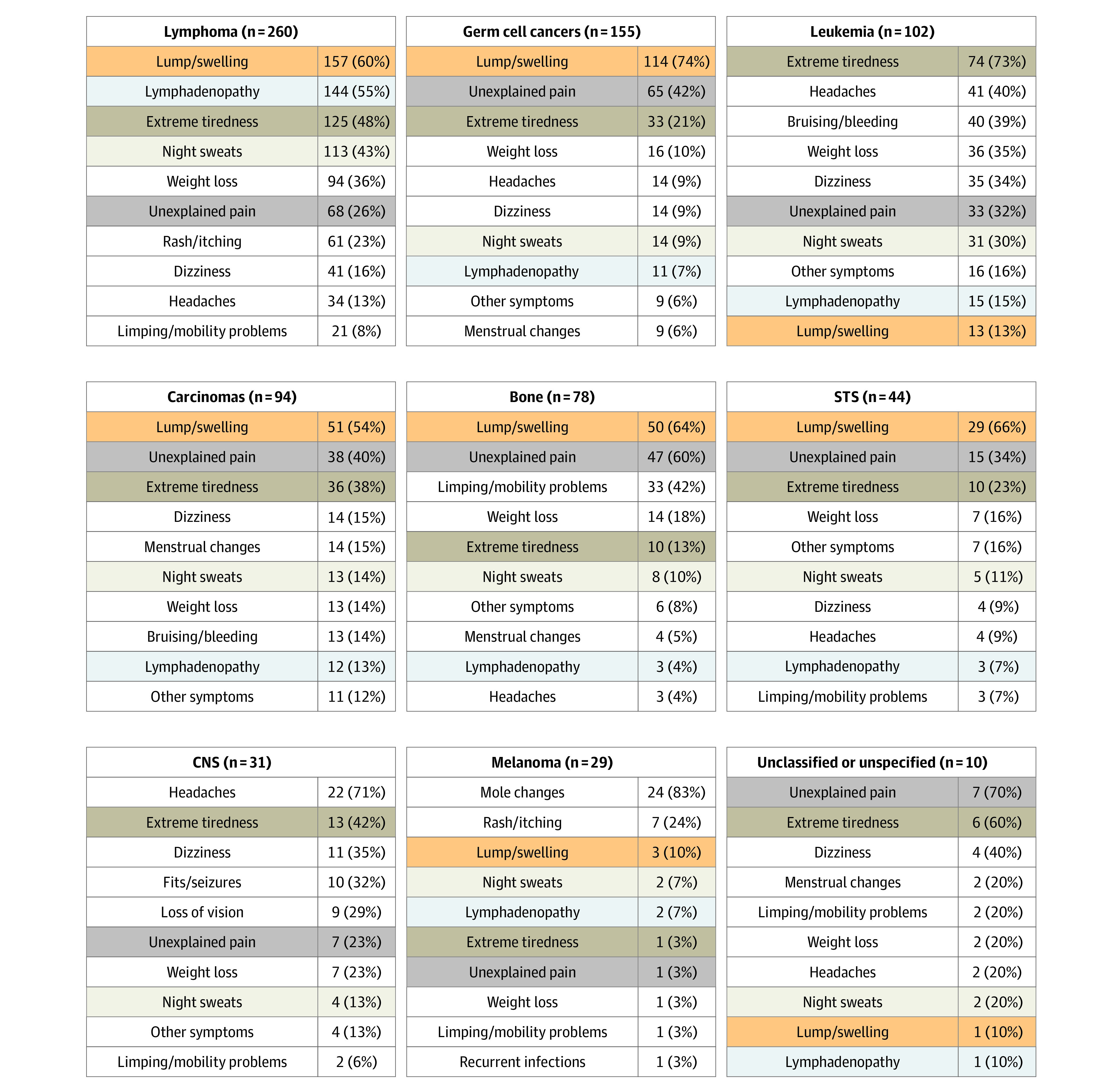
Frequencies of the 10 Most Common Symptoms Among Adolescent and Young Adults by Cancer Group The 5 most common symptoms across the cohort are highlighted across cancer groups by color. Germ cell cancers comprise 119 patients with testicular germ cell cancer (77%), 11 patients with ovarian germ cell cancer (7%), and 25 patients with other germ cell cancers (16%). See eTable 2 in the Supplement for full symptom signatures. CNS indicates central nervous system and other intracranial and intraspinal neoplasms; STS, soft-tissue sarcomas.

The symptom signature of presenting symptoms varied by cancer group (Figure 2 and eTable 2 in the Supplement). Some symptoms were highly prevalent across several cancer groups; for example, lump or swelling was reported in more than 50% of patients with lymphomas, germ cell cancers, carcinomas, bone tumors, and soft-tissue sarcomas, and unexplained pain was reported by at least 25% of patients in all cancer groups apart from melanoma (1 patient [3%]) and central nervous system tumors (7 patients [23%]). In contrast, some symptoms tended to be cancer specific (present in at least one-quarter of adolescent and young adults with those cancers); for example, limping or mobility problems were present in 33 patients with bone tumors (42%), bruising or bleeding were present in 40 patients with leukemia (39%), and fits or seizures (10 patients [32%]) and loss of vision (9 patients [29%]) were present among patients with central nervous system tumors.

Extreme tiredness was 1 of the 5 most frequent symptoms experienced before diagnosis among 8 of 9 cancer groups, ranging from 10 patients with bone tumors (13%) to 74 patients (73%) with leukemia. Night sweats was 1 of the 10 most frequent symptoms listed for all 9 cancer groups.

### Presenting Symptoms and the Patient Interval

Almost one-half (385 patients [48%]) of the 745 patients with complete information on the patient interval had a patient interval longer than 2 weeks, 204 patients (27%) had an interval longer than 1 month, and 91 patients (12%) longer than 3 months. The proportion of patients with patient intervals longer than 1 month varied by presenting symptom, ranging from 6% (1 patient) for fits or seizures to 43% (18 patients) for recurrent infections, with variation by cancer site (see eTable 3 in the Supplement for proportion of patients who had a patient interval >1 month by symptom, stratified by cancer site).

## Discussion

In a nationwide cohort of adolescents and young adults with cancer, most patients reported multiple symptoms before diagnosis. Lump or swelling was the most commonly reported presenting symptom, followed by extreme tiredness and unexplained pain. The nature of presenting symptoms varied by cancer group, but there were symptoms common across all cancer types. More than one-quarter (27%) of adolescent and young adults with cancer presented more than 1 month after symptom onset, with some observed variation between presenting symptoms.

Literature on the nature and frequency of symptoms experienced by adolescents and young adults before cancer diagnosis is sparse; study populations often exclude those older than 16 years.^20,25,26^ Most existing studies^6,19,27^ on adolescents and young adults are based on information captured in EHRs. In comparison, our findings based on self-reported data indicate substantially higher frequencies of presenting symptoms among adolescent and young adults before cancer diagnosis. This is in line with previously observed disparities between self-reported and EHR-based symptom frequencies among adult populations.^28,29,30^

Regarding associations between symptoms and diagnostic timeliness, a previous study^26^ that examined 3 categories of symptoms (pain, growing swelling or mass, or “specific symptoms”) among primarily older children and adolescents (ie, aged ≥10 years) with solid tumors found no statistically significant evidence for variation in time to help-seeking. In our study, the sample size precluded the use of multivariable regression to examine differences in patient interval between individual symptoms while adjusting for potential confounders.^31^ Nonetheless, our observed (crude) findings are compatible with those reported by Veneroni and colleagues^26^; namely, the proportion of patients with intervals longer than a month was broadly similar between different presenting symptoms.

Our findings show that adolescents and young adults with cancer present with a higher frequency of symptoms than previously estimated. Furthermore, although some cancer types have relatively distinct symptom signatures dominated by a single symptom (eg, mole or skin changes and melanoma), many presenting symptoms are shared across multiple cancer types and are often vague and nonspecific in nature, such as extreme tiredness, unexplained pain, and night sweats.

Understanding the presenting symptoms of adolescents and young adults with cancer could inform the design of interventions to raise awareness of cancer symptoms. Although we described variation in the timeliness of help-seeking among the 17 studied symptoms, future (larger) studies could examine this accounting for patient characteristics and cancer group.

Our results indicate substantially higher prevalence of presenting symptoms among adolescents and young adults with cancer compared with previous research based on structured items (coded entries) in EHR data.^19^ For example, lump or mass has previously been reported as being present in 24% of patients with lymphoma,^19^ whereas patient-reported data in the present study indicate a frequency of more than 50%; similarly, fatigue has been reported as being present in 11% of patients with leukemia^19^ compared with a frequency of 73% in our data. These observations suggest that substantial underestimation of symptoms among adolescents and young adults with cancer is likely when using information from structured fields in EHRs, because of incompletely coded entries or incomplete reporting of symptoms to the general practitioner by adolescents and young adults.

It is worth considering the potential implications of the underestimation of true symptom frequency on previously reported positive predictive values (PPVs) of presenting symptoms.^19^ If the degree of undercoding of symptoms is differential between cases and controls, then this would bias the estimation of the PPV. Specifically, greater undercoding in cases more than controls would affect the true-positive and false-negative counts, and lead to underestimation of the PPV. In comparison, greater undercoding in controls more than cases would affect false-positive and true-negative counts, leading to overestimation of the PPV. Further work examining the prevalence of presenting symptoms among adolescents and young adults with and without cancer is, therefore, merited. Use of optimized EHRs, including free-text information (eg, decision-support systems),^32^ or combining both EHR-derived information and self-reported data may be informative.^33,34^

### Strengths and Limitations

To our knowledge, this is the largest study to date to examine the self-reported symptoms of adolescents and young adults before cancer diagnosis. Our findings are based on a large cohort of adolescent and young adult patients with cancer, encompassing self-reported data on both symptoms and timeliness of help-seeking. Survey participants were largely representative of incident cancer cases, with some underrepresentation of brain tumors, melanoma, and carcinomas.^22^

Although the BRIGHTLIGHT cohort represents one of the largest adolescent and young adult study populations with diagnostic pathway information, the broad spectrum of presenting symptoms in the cohort meant that analyses adjusting for potential confounders of interest, such as patient characteristics and cancer group, were not possible. Specifically, the number of patients with the outcome of interest (ie, a patient interval of >1 month) relative to the number of exposures of interest (17 symptom categories) was too small for adjusted analyses.^31^

Self-reported information on symptoms and intervals to help-seeking collected retrospectively is associated with risk of recall bias, and a priori excludes patients with poor prognosis who may die soon after their diagnosis, leading to survival bias. We observed much higher estimates of symptom prevalence before diagnosis than those previously estimated using data from EHRs, which are prone to underrecording of symptoms and their duration because of incomplete disclosure or elicitation during the consultation, and/or incomplete entering of information in the patient’s EHR, particularly if only coded entries are used for research.^21,33,35^

## Conclusions

Adolescents and young adults with cancer present with a broad spectrum of symptoms, some of which are common across different cancer groups. The degree of observed variation in time to help-seeking by presenting symptom is not large enough to enable specific targeting within awareness campaigns. Our findings indicate substantially greater frequencies of presenting symptoms among adolescents and young adults with cancer than previously reported; re-evaluation of their prevalence and PPV is warranted.

## Notes

Supplement. **eTable 1.** Presenting Symptoms Among the BRIGHTLIGHT Cohort **eFigure.** Flow Diagram Indicating Derivation of the Study Population **eTable 2.** Cancer-Specific Symptom Signatures (Frequency of Symptoms by Cancer Site) **eTable 3.** Percentages of a Patient Interval >1 Month Associated With Each Symptom, Stratified by Cancer Site
