# Supplementary material for: Association of Self-reported Presenting Symptoms With Timeliness of Help-Seeking Among Adolescents and Young Adults With Cancer in the BRIGHTLIGHT Study
Source: JAMA Netw Open. 2020 Sep 3;3(9):e2015437. doi: 10.1001/jamanetworkopen.2020.15437 (PMC7489839; doi:10.1001/jamanetworkopen.2020.15437)
Supplement: Supplement. — eTable 1. Presenting Symptoms Among the BRIGHTLIGHT Cohort eFigure. Flow Diagram Indicating Derivation of the Study Population eTable 2. Cancer-Specific Symptom Signatures (Frequency of Symptoms by Cancer Site) eTable 3. Percentages of a Patient Interval >1 Month Associated With Each Symptom, Stratified by Cancer Site [file jamanetwopen-e2015437-s001.pdf]

Supplementary Online Content

Koo MM, Lyratzopoulos G, Herbert A, et al. Association of self-reported presenting symptoms with timeliness of help-seeking among adolescents and young adults with cancer in the BRIGHTLIGHT study. *JAMA Netw Open*. 2020;3(9):e2015437. doi:10.1001/jamanetworkopen.2020.15437

eTable 1. Presenting Symptoms Among the BRIGHTLIGHT Cohort

eFigure. Flow Diagram Indicating Derivation of the Study Population

eTable 2. Cancer-Specific Symptom Signatures (Frequency of Symptoms by Cancer Site)

eTable 3. Percentages of a Patient Interval >1 Month Associated With Each Symptom, Stratified by Cancer Site

This supplementary material has been provided by the authors to give readers additional information about their work.

eTable 1: Presenting symptoms among the BRIGHTLIGHT cohort

Elicited as part of the BRIGHTLIGHT survey on pre-diagnostic events with the following question: "Did you have any of the symptoms listed on this card? Please just read out the letter or letters that apply."

**Symptom included in the current analysis**	**Details of provenance**
Unexplained pain	Originally described as "Pain for no apparent reason"
Lump/swelling	Originally described as "Lump or swelling"
Lymphadenopathy	Originally described as "Swollen/enlarged lymph nodes (Your doctor may have called this lymphadenopathy)"
Headaches	Originally described as "Headaches for no apparent reason"
Extreme tiredness	Originally described as "Extreme tiredness (your doctor may have called this fatigue)"
Weight loss	Originally described as "Losing weight/loss of weight without dieting"
Bruising/bleeding	Originally described as "Bruising or bleeding for no apparent reason"
Loss of vision	Originally described as "Loss of vision"
Mole changes	Originally described as "Change in the size, colour or appearance of a mole"
Menstrual changes	Originally described as "Changes in your periods" [only asked to participants coded as female
Dizziness	Originally described as "Feeling dizzy"
Fits/seizures	Originally described as "Having fits/seizures"
Limping/mobility	Originally described as "Limping/mobility problems"
Rash/itching	Originally described as "Rash/itching"
Recurrent infections	Originally described as "Recurrent infections"
Night sweats	Originally described as "Sweating at night"
Other symptoms	Originally described as "Other symptoms (please state)", encompassing a range of free-text responses including the following:<br>CNS/PNS symptoms<br>Breathlessness<br>Nausea/vomiting<br>Cough<br>Fever<br>Fainting/pallor<br>Oedema<br>ENT symptoms<br>Haematuria<br>Haemoptysis

CNS=central nervous system; ENT=ear, nose, throat related; PNS=peripheral nervous system.

© 2020 Koo MM et al. *JAMA Network Open.*

eFigure. Flow Diagram Indicating Derivation of the Study Population

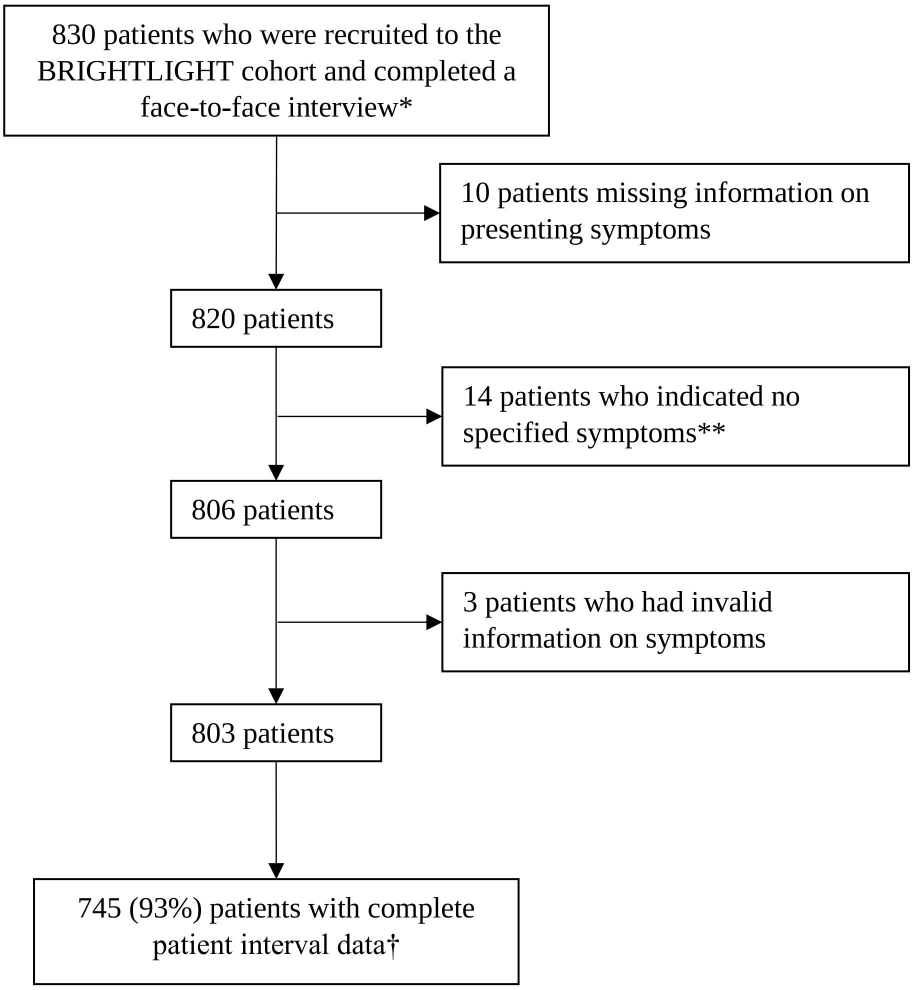

* BRIGHTLIGHT aimed to recruit 13–24 year olds, however five patients aged 12 years were included in the study due to mismatched dates of diagnosis between the recruiting centre and cancer registration (these patients were included as they were close to their 13th birthday at recruitment).

**The 14 patients who had not specified symptoms were more likely to be female, married/in a civil partnership, and subsequently diagnosed with melanomas or soft-tissue sarcomas.

† For patients who had missing date of symptom onset or date of help-seeking, the patient interval (time from symptom onset to first clinical presentation) could not be estimated (Herbert et al., 2018 http://dx.doi.org/10.1016/S2352-4642(18)30004-X).

© 2020 Koo MM et al. *JAMA Network Open.*

eTable 2: Cancer-Specific Symptom Signatures (Frequency of Symptoms by Cancer Site)

The frequency of symptoms by cancer site (calculated by column). As the majority of patients had multiple symptoms, the proportions do not sum to 100%.

Symptom	Lymphoma (n=260)	Germ cell[1] (n=155)	Leukaemia (n=102)	Carcinomas (n=94)	Bone (n=78)	STS (n=44)	CNS (n=31)	Melanoma (n=29)	Unclass. or Unspec. (n=10)
Lump/swelling	60%	74%	13%	54%	64%	66%	3%	10%	10%
Extreme tiredness	48%	21%	73%	38%	13%	23%	42%	3%	60%
Unexplained pain	26%	42%	32%	40%	60%	34%	23%	3%	70%
Night sweats	43%	9%	30%	14%	10%	11%	13%	7%	20%
Lymphadenopathy	55%	7%	15%	13%	4%	7%	.	7%	10%
Weight loss	36%	10%	35%	14%	18%	16%	23%	3%	20%
Headaches	13%	9%	40%	7%	4%	9%	71%	.	20%
Dizziness	16%	9%	34%	15%	4%	9%	35%	.	40%
Rash/itching	23%	2%	12%	5%	4%	2%	3%	24%	10%
Limping/mobility problems	8%	1%	10%	3%	42%	7%	6%	3%	20%
Recurrent infections	7%	3%	12%	10%	3%	.	.	3%	.
Other symptoms	6%	6%	16%	12%	8%	16%	13%	.	10%
Bruising/bleeding	4%	3%	39%	14%	1%	2%	3%	.	10%
Menstrual changes	5%	6%	10%	15%	5%	2%	3%	.	20%
Mole changes	2%	.	5%	2%	3%	2%	.	83%	.
Fits/seizures	2%	.	.	2%	1%	.	32%	.	.
Loss of vision	1%	1%	9%	2%	1%	5%	29%	.	.

CNS=CNS and other intracranial and intra-spinal neoplasms; STS=soft tissue sarcomas; Unclass. / unspec. = unclassified/ unspecified

[1] Comprising patients with testicular germ cell cancer (n=119, 77%), ovarian germ cell cancer (n=11, 7%), and other germ cell tumours (n=25, 16%)

© 2020 Koo MM et al. *JAMA Network Open.*

eTable 3: Percentages of a Patient Interval >1 Month Associated With Each Symptom, Stratified by Cancer Site

The percentages in the table below indicate the proportion of patients with a patient interval >1 month among all patients with a particular presenting symptom. For example, of the 149 lymphoma patients who presented with lump/swelling, 40 (27%) had a patient interval >1 month; while of the 117 lymphoma patients who presented with extreme tiredness, 35 (30%) of patients had a patient interval >1 month.

Presenting symptom	Proportion of cancer patients who had a patient interval >1 month *Based on 745 patients with complete patient interval information* [1]								
	Lymphoma	Germ cell[2]	Leukaemia	Carcinomas	Bone	STS	CNS	Melanoma	Unclass. or Unspec.
Total (any symptom)	66/248 (27%)	36/146 (25%)	22/88 (25%)	28/87 (32%)	17/68 (25%)	13/41 (32%)	5/29 (17%)	14/28 (50%)	3/10 (30%)
Lump/swelling	40/149 (27%)	25/108 (23%)	4/11 (36%)	13/47 (28%)	9/44 (20%)	9/27 (33%)	1/1 (100%)	2/3 (67%)	0/1 (0%)
Extreme tiredness	35/117 (30%)	9/30 (30%)	15/62 (24%)	10/33 (30%)	4/10 (40%)	4/10 (40%)	3/13 (23%)	1/1 (100%)	2/6 (33%)
Unexplained pain	17/66 (26%)	17/61 (28%)	10/29 (34%)	10/34 (29%)	13/40 (33%)	5/15 (33%)	1/7 (14%)	1/1 (100%)	3/7 (43%)
Night sweats	37/108 (34%)	7/13 (54%)	9/26 (35%)	4/13 (31%)	4/8 (50%)	2/4 (50%)	0/4 (0%)	1/1 (100%)	0/2 (0%)
Lymphadenopathy	40/136 (29%)	1/11 (9%)	3/13 (23%)	4/12 (33%)	1/3 (33%)	0/3 (0%)		1/2 (50%)	0/1 (0%)
Weight loss	30/89 (34%)	5/15 (33%)	11/33 (33%)	4/11 (36%)	3/13 (23%)	3/7 (43%)	1/6 (17%)	0/1 (0%)	0/2 (0%)
Headaches	12/31 (39%)	6/12 (50%)	9/36 (25%)	1/5 (20%)	3/3 (100%)	1/4 (25%)	4/21 (19%)		0/2 (0%)
Dizziness	11/39 (28%)	5/12 (42%)	7/31 (23%)	3/13 (23%)	2/3 (67%)	2/4 (50%)	2/11 (18%)		0/4 (0%)
Rash/itching	14/59 (24%)	1/2 (50%)	1/9 (11%)	1/4 (25%)	1/3 (33%)	1/1 (100%)	0/1 (0%)	5/7 (71%)	0/1 (0%)
Limping/mobility problems	4/17 (24%)	0/2 (0%)	2/10 (20%)	0/3 (0%)	9/29 (31%)	2/3 (67%)	1/2 (50%)	0/1 (0%)	0/2 (0%)
Recurrent infections	8/18 (44%)	1/3 (33%)	1/9 (11%)	6/9 (67%)	1/2 (50%)			1/1 (100%)	
Other symptoms	4/15 (27%)	5/9 (56%)	3/16 (19%)	3/11 (27%)	1/3 (33%)	1/7 (14%)	0/4 (0%)		0/1 (0%)
Bruising/bleeding	1/10 (10%)	2/4 (50%)	8/33 (24%)	5/12 (42%)	1/1 (100%)		0/1 (0%)		0/1 (0%)
Menstrual changes	1/12 (8%)	2/8 (25%)	1/8 (13%)	6/12 (50%)	2/3 (67%)	1/1 (100%)	0/1 (0%)		1/2 (50%)
Mole changes	0/6 (0%)		1/5 (20%)	1/2 (50%)	0/2 (0%)	1/1 (100%)		12/24 (50%)	
Fits/seizures	0/4 (0%)			0/2 (0%)	0/1 (0%)		1/10 (10%)		
Loss of vision	1/3 (33%)		2/6 (33%)	0/1 (0%)	0/1 (0%)	1/2 (50%)	4/8 (50%)		

CNS=CNS and other intracranial and intra-spinal neoplasms; STS=soft tissue sarcomas; Unclass. / unspec. = unclassified/ unspecified

[1] Numerators of the presented proportions in each column do not sum to the total number of patients with a patient interval >1 month as indicated in the first row, as some patients had multiple presenting symptoms.

[2] Comprising patients with testicular germ cell cancer (n=119, 77%), ovarian germ cell cancer (n=11, 7%), and other germ cell tumours (n=25, 16%)

Blank cells indicate that there were no patients that presented with a specific symptom (row) and were diagnosed with a specific cancer (column), while zero percentages indicate that all patients with that particular symptom/cancer pair had a patient interval ≤1 month.

© 2020 Koo MM et al. *JAMA Network Open.*
